# Idiopathic Intracranial Hypertension Without Papilledema in a Transgender Woman With HIV: A Case Report and Review of Risk Factors

**DOI:** 10.7759/cureus.105116

**Published:** 2026-03-12

**Authors:** Susana P Garcia, Peter Hsin, Shon Shmushkevich, Magalys L Suarez, Lorena Bonilla

**Affiliations:** 1 Internal Medicine, Florida International University, Herbert Wertheim College of Medicine, Miami, USA; 2 Family Medicine, Baptist Health South Florida, Miami, USA

**Keywords:** antiretroviral therapy, biktarvy, estrogen therapy, hiv, idiopathic intracranial hypertension, intracranial pressure, neuro-ophthalmology, papilledema, transgender woman, visual field loss

## Abstract

Idiopathic intracranial hypertension (IIH) is a disorder characterized by elevated intracranial pressure (ICP) in the absence of intracranial mass lesions, hydrocephalus, or infection. The condition most commonly presents with headache and papilledema; however, idiopathic intracranial hypertension without papilledema (IIHWOP) is increasingly recognized as a clinically important variant that may lead to diagnostic delay and progressive visual impairment. Established risk factors include obesity, female sex, and hormonal influences, while IIH in people living with HIV remains uncommon. We report a rare case of IIHWOP in a transgender woman with HIV receiving bictegravir/emtricitabine/tenofovir alafenamide (Biktarvy) and estrogen therapy.

A 34-year-old transgender woman with well-controlled HIV, assigned male at birth, presented with six months of progressive bilateral peripheral visual field loss accompanied by chronic headache, nausea, dizziness, and diplopia. Neurologic examination revealed severe visual field constriction without papilledema. Neuroimaging demonstrated findings suggestive of chronically elevated ICP, including prominent Meckel’s caves and mild flattening of the superior pituitary margin, without evidence of mass lesions or venous sinus thrombosis. Lumbar puncture revealed a markedly elevated opening pressure of 53 cm H₂O with otherwise normal cerebrospinal fluid composition. Infectious, inflammatory, and neoplastic etiologies were excluded. The patient was diagnosed with IIHWOP and treated with acetazolamide, with plans for close neuro-ophthalmologic follow-up.

This case underscores the importance of considering IIH variants in patients with progressive visual symptoms despite a normal funduscopic examination. The potential contributions of antiretroviral therapy, estrogen use, obesity, and psychosocial stressors warrant further investigation.

## Introduction

Idiopathic intracranial hypertension (IIH) is defined by elevated intracranial pressure (ICP) with normal cerebrospinal fluid composition and no identifiable structural intracranial pathology on neuroimaging [1]. The disorder predominantly affects women of childbearing age and has a strong association with obesity [2]. Typical clinical features include headache, transient visual obscurations, pulsatile tinnitus, and diplopia, with papilledema traditionally regarded as a key diagnostic finding [3].

However, idiopathic intracranial hypertension without papilledema (IIHWOP) has gained increasing recognition as a clinically important variant [4]. Patients with IIHWOP may present with headache and visual field deficits despite the absence of optic disc edema, leading to diagnostic delay and a potential risk of irreversible visual impairment. Despite growing recognition, IIHWOP remains underrecognized in clinical practice, particularly when visual symptoms occur without classic funduscopic findings. Additionally, IIH in people living with HIV is rare, and the relationship between contemporary antiretroviral therapy and intracranial hypertension is not well defined [5]. Reports describing IIH in HIV-positive individuals remain limited, and there is a paucity of literature examining the potential association between integrase strand transfer inhibitors, including bictegravir, and elevated ICP.

Given the rarity of IIH in people living with HIV and the absence of papilledema in some patients, recognition of IIHWOP can be challenging. The coexistence of HIV infection, antiretroviral therapy, gender-affirming estrogen use, and obesity represents a clinical overlap that has been rarely described in the literature. Here, we present a case of a transgender woman with HIV receiving antiretroviral therapy and gender-affirming hormone therapy that highlights these overlapping risk factors and underscores the need for heightened clinical suspicion in similarly complex presentations.

## Case presentation

A 34-year-old transgender woman with a history of HIV diagnosed in 2017, obesity, prior treated syphilis, and gender-affirming surgery presented with progressive bilateral peripheral visual field loss over six months. The patient endorsed chronic right-sided headache radiating to the right eye, nausea, dizziness, and intermittent horizontal diplopia. She denied substance use, smoking, recent travel, or systemic infectious symptoms.

At the time of presentation, the patient was adherent to her medication regimen, including bictegravir/emtricitabine/tenofovir alafenamide (Biktarvy), which had been initiated one month prior following transition from Triumeq, and estrogen therapy for gender-affirming care. Given that her visual symptoms had been ongoing for six months, symptom onset predated initiation of Biktarvy. Her HIV infection was well controlled, with a CD4 count of 844 cells/µL (reference range: 500-1,200 cells/µL) at 28% (reference range: 30-60%) and low-level viremia. There was no history of opportunistic infections. Psychosocial history was notable for prior human trafficking and ongoing psychosocial stressors, which were considered in the broader clinical context.

Physical examination results

On presentation, vital signs demonstrated hypertension with a blood pressure of 149/100 mmHg. The patient was afebrile and saturating 98% on room air. General examination revealed an obese woman (BMI 36.7 kg/m²) in no acute distress, alert and fully oriented.

Confrontation visual field testing demonstrated severe bilateral concentric peripheral field constriction with preservation of central vision, consistent with bitemporal hemianopia. Color vision testing revealed red and green desaturation. She exhibited gait instability attributed to visual impairment. Cranial nerve examination demonstrated deficits involving cranial nerve VIII (left-sided hearing loss) and cranial nerves III, IV, and VI (horizontal diplopia), with no ptosis or nystagmus. Motor strength and sensation were intact, and no focal deficits were identified. Funduscopic examination revealed no papilledema. Cardiovascular and respiratory examinations were unremarkable.

Laboratory results and imaging 

Basic metabolic panel revealed mild hyperglycemia (165 mg/dL; reference range: 70-100 mg/dL, fasting) and low calcium (8.3 mg/dL; reference range: 8.5-10.5 mg/dL), with a hemoglobin A1c of 5.8% (reference range: <5.7% normal, 5.7-6.4% prediabetes). Inflammatory markers were within normal limits, including a C-reactive protein level <2.9 mg/L (reference range: <3.0 mg/L) and an erythrocyte sedimentation rate of 6 mm/hr (reference range: 0-20 mm/hr for men, 0-30 mm/hr for women). Infectious workup demonstrated a reactive treponemal antibody with a non-reactive rapid plasma reagin, findings consistent with previously treated syphilis. Serologic testing for toxoplasmosis and acute cytomegalovirus infection was negative.

Cerebrospinal fluid analysis showed normal glucose (reference range: 50-80 mg/dL or 2.8-4.4 mmol/L) and protein levels (reference range: 15-45 mg/dL), absent xanthochromia, and a differential consisting of 57% lymphocytes (reference range: 40-80%), 29% monocytes (reference range: 15-45%), and 14% neutrophils (reference range: 0-6%), without evidence of infection. Cryptococcal antigen testing subsequently returned negative. Lumbar puncture revealed a markedly elevated opening pressure of 53 cm H₂O, consistent with intracranial hypertension.

**Figure 1:**
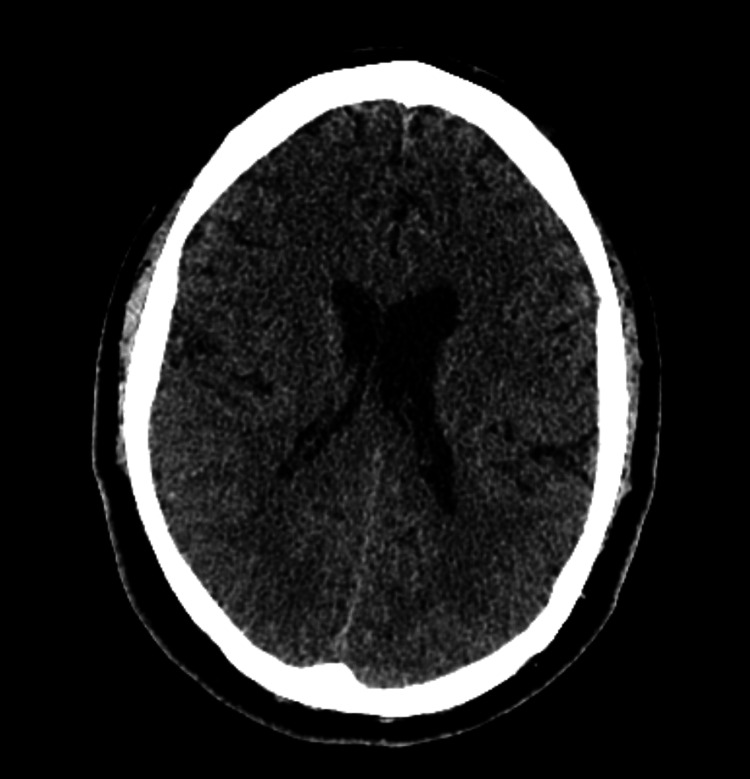
Non-contrast CT head demonstrating no acute intracranial process. Axial images show no evidence of territorial infarct, intracranial hemorrhage, mass effect, midline shift, or hydrocephalus. Ventricular size is within normal limits, and basal cisterns are patent. Paranasal sinuses and mastoid air cells appear clear.

**Figure 2:**
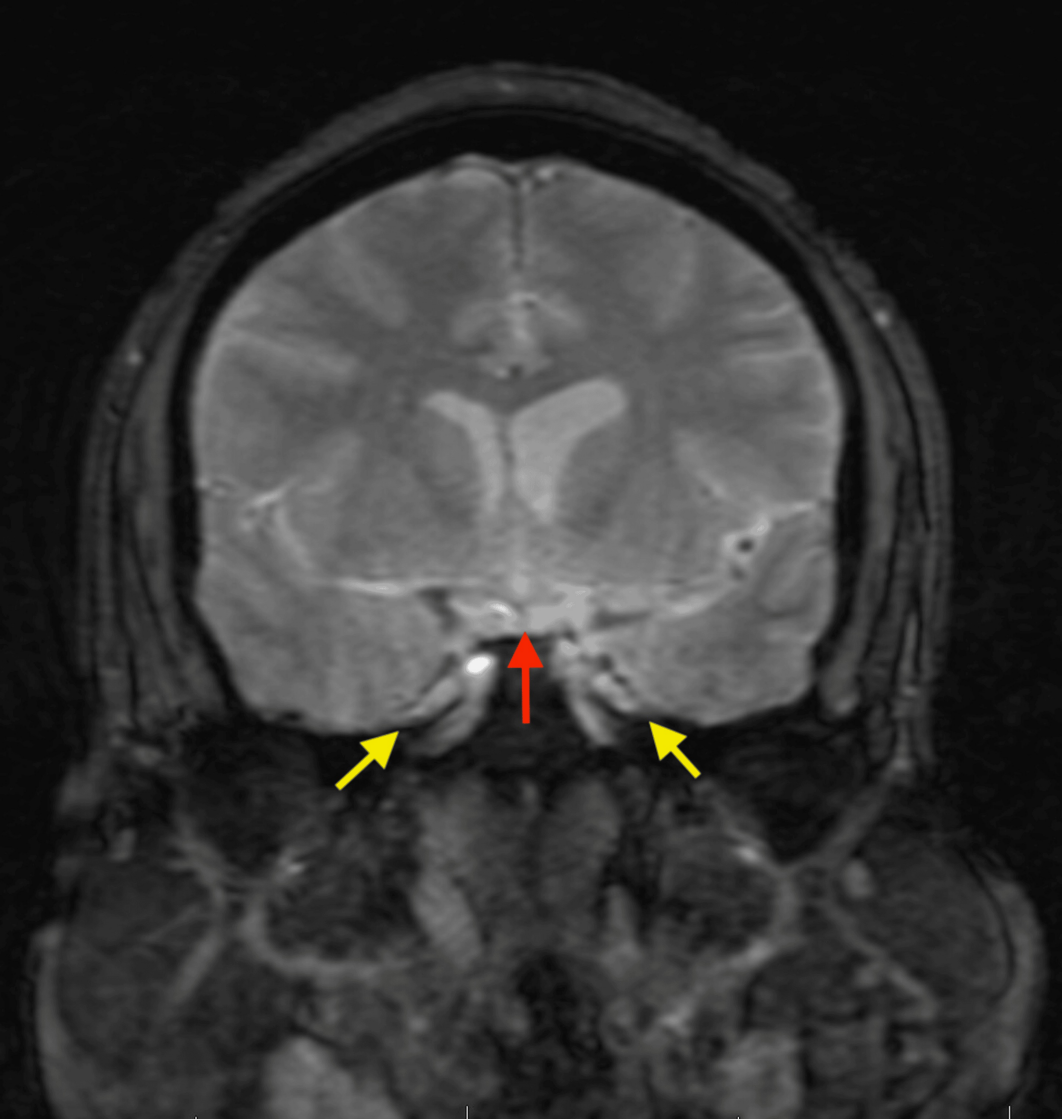
Non-contrast MRI brain demonstrating findings suggestive of elevated intracranial pressure. Multiplanar T2-weighted sequences show symmetrically prominent Meckel’s caves (yellow arrows) with mild flattening of the superior pituitary margin (red arrow), changes associated with chronically raised intracranial pressure.

**Figure 3:**
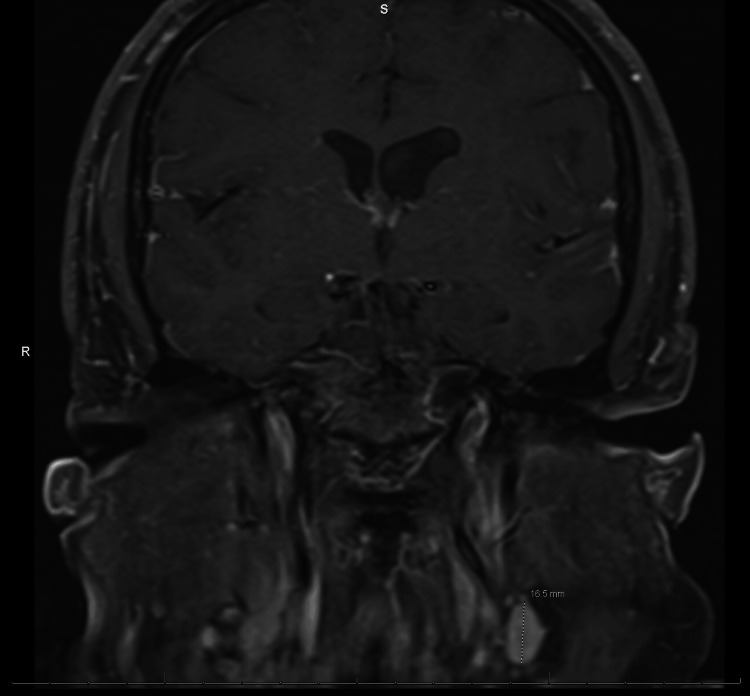
MRI brain and orbits with contrast demonstrating no abnormal enhancement or intracranial mass. Postcontrast sequences show preserved optic nerve sheath complexes and normal pituitary morphology without enhancement of the brain, optic nerves, or orbital structures.

**Figure 4:**
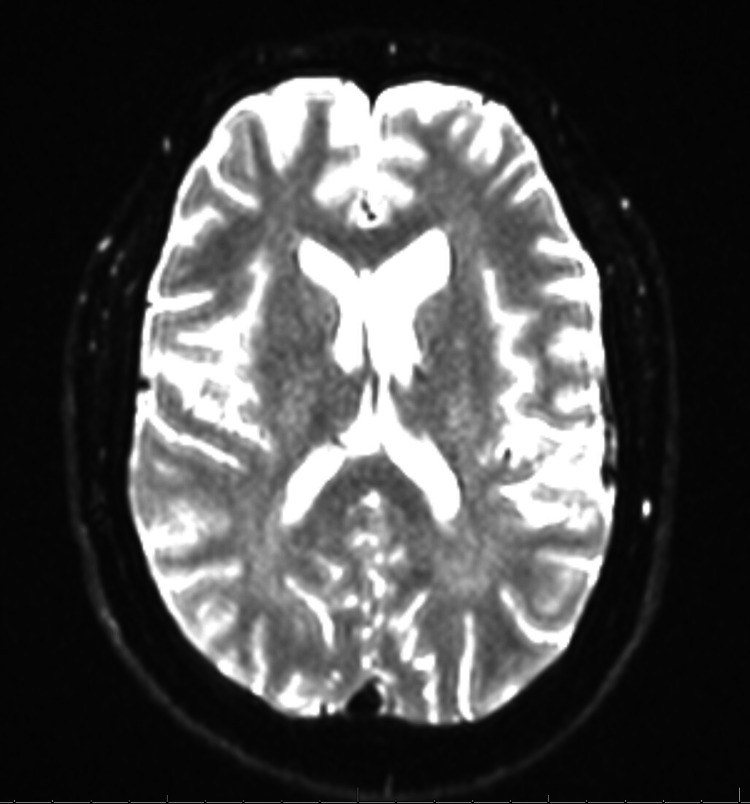
MR venography showing no evidence of venous sinus thrombosis. The dural venous systems are patent, with a hypoplastic left transverse sinus identified as a normal variant.

Neuroimaging was performed to evaluate for structural, inflammatory, or vascular causes of the patient’s symptoms. Figure 1, a non-contrast CT of the head, demonstrates no acute intracranial pathology. Figure 2, a non-contrast MRI, shows imaging features consistent with chronically elevated ICP, including prominent Meckel’s caves and flattening of the superior pituitary margin. Figure 3, a contrast-enhanced MRI of the brain and orbits, reveals no abnormal enhancement or mass lesions. Figure 4, MR venography, demonstrates patent dural venous sinuses without evidence of venous sinus thrombosis.

## Discussion

This case underscores the diagnostic complexity of IIHWOP in patients with overlapping hormonal and infectious risk factors and raises consideration of a possible association with contemporary antiretroviral and estrogen therapy. Although papilledema has traditionally been considered central to the diagnosis of IIH, accumulating evidence supports the existence of IIHWOP as a clinically significant variant [4]. Patients with IIHWOP may present predominantly with headache and visual field deficits, placing them at risks of delayed diagnosis and permanent visual impairment.

The differential diagnosis in this patient included compressive optic neuropathy, optic neuritis, cavernous sinus thrombosis, skull base osteomyelitis, infectious meningitis, and HIV-related opportunistic infections. Extensive neuroimaging excluded mass lesions and venous sinus thrombosis, while CSF analysis ruled out infectious and inflammatory etiologies. MRI findings of prominent Meckel’s caves and flattening of the superior pituitary margin supported chronically elevated ICP [6].

IIH is increasingly conceptualized as a disorder of ICP homeostasis in which CSF production, CSF outflow resistance, and cerebral venous pressures interact in a self-reinforcing feedback loop, most strongly influenced at the population level by obesity and recent weight gain [7]. Obesity is a well-established risk factor for IIH, and estrogen therapy has been implicated in altered CSF dynamics and increased ICP [2,8]. Estrogen receptors (ERα and ERβ) have been identified in human choroid plexus epithelium, supporting biologic plausibility for steroid-mediated modulation of CSF transport mechanisms. Experimental models suggest that sex steroids can influence sodium transport and Na⁺/K⁺-ATPase activity within the choroid plexus, although direct human evidence linking exogenous estrogen exposure to sustained increases in ICP remains limited and frequently confounded by body mass index and weight gain [9].

The role of antiretroviral therapy remains less clear. While older agents have been associated with intracranial hypertension, data regarding integrase inhibitor-based regimens are limited [10]. Isolated case reports have described IIH in patients receiving bictegravir-containing therapy, though causality has not been established [11]. Integrase strand transfer inhibitors (INSTIs) are associated with weight gain in randomized and observational studies. Given the strong link between recent weight gain and IIH risk, the most plausible mechanism connecting INSTIs to intracranial hypertension is indirect, via adiposity-related increases in venous pressure and impaired CSF resorption, rather than a direct effect on CSF production [12]. Given the recent initiation of Biktarvy and the lack of definitive evidence, antiretroviral therapy was continued.

Psychosocial stressors and prior trauma may have contributed indirectly through stress-related weight gain and metabolic dysregulation, further compounding established risk factors for IIH [13].

Based on current evidence, obesity and recent weight gain likely represent the dominant contributors in this case, with estrogen exposure serving as a potential additive factor. The contribution of integrase inhibitor-based therapy remains uncertain and most plausibly indirect via weight gain, while psychosocial stressors are best understood as modifiers rather than primary etiologic drivers.

Published reports of IIH in transgender women remain limited and are largely confined to individual case reports and small case series [14-16]. While IIH has been described in people living with HIV [17-19] and intracranial hypertension has been reported in transgender patients receiving gender-affirming hormone therapy (including estrogen exposure) [15,16], documented cases in which HIV infection, integrase inhibitor-based antiretroviral therapy, obesity, and concurrent estrogen exposure converge within a single patient appear to be exceedingly rare in the available literature [15]. Notably, in the most comparable published case of IIH in a transgender woman with well-controlled HIV receiving an integrase inhibitor-containing regimen, prior estrogen use was remote rather than ongoing at presentation (estimated discontinuation four to six years earlier) [15], underscoring the diagnostic complexity and challenges of causal attribution when multiple potential risk factors coexist.

Management of IIH focuses on reduction of ICP and preservation of visual function. Acetazolamide remains first-line therapy and has demonstrated benefits in improving visual outcomes [20]. Acetazolamide was selected in this case given its established efficacy in reducing CSF production and its role as a standard first-line medical therapy. Alternative agents such as topiramate were considered; however, given the patient’s predominant visual symptoms and absence of medication intolerance, acetazolamide was initiated. Surgical interventions were not indicated due to the absence of fulminant visual decline or failure of medical therapy.

Beyond pharmacologic therapy, weight management plays a critical role in long-term disease control. Observational studies have shown improvement in symptoms and visual outcomes following sustained weight reduction [21], while interventional studies demonstrate that caloric restriction can significantly reduce ICP [22]. The patient was counseled on weight management strategies, and ongoing monitoring of estrogen therapy was recommended in coordination with her gender-affirming care team.

Recognition and early diagnosis of IIH, particularly in atypical cases without papilledema, are essential. Expert recommendations emphasize maintaining a high index of suspicion in patients with chronic headache and visual field abnormalities and advocate for prompt neuroimaging and lumbar puncture when IIH is suspected [23,24].

## Conclusions

The absence of papilledema does not exclude intracranial hypertension; clinicians should maintain a high index of suspicion for IIHWOP in high-risk patients, including those with obesity, HIV infection, and exposure to hormone therapy, who present with chronic headache or progressive visual disturbances. In such patients, comprehensive evaluation, including neuroimaging, lumbar puncture, and formal visual field assessment, when indicated, is essential to exclude secondary causes and confirm elevated ICP.

In this patient, the etiology was likely multifactorial, with obesity and recent weight trajectory representing the most established contributors, estrogen exposure serving as a potential additive factor, and the contribution of contemporary antiretroviral therapy remaining uncertain. Early recognition and timely management are critical to preserving visual function and optimizing outcomes.

Prospective studies are needed to better define the relationship between integrase inhibitor-based regimens, weight gain, and IIH risk. For transgender patients receiving gender-affirming hormone therapy in the setting of obesity, periodic assessment of weight trajectory and structured review of headache and visual symptoms may facilitate earlier detection of intracranial hypertension.
